# Two New Biocontrol Agents Against Clubroot Caused by *Plasmodiophora brassicae*

**DOI:** 10.3389/fmicb.2019.03099

**Published:** 2020-01-21

**Authors:** Manli Zhu, Youwei He, Yi Li, Tirong Ren, Hao Liu, Junbin Huang, Daohong Jiang, Tom Hsiang, Lu Zheng

**Affiliations:** ^1^The Key Lab of Plant Pathology of Hubei Province, Huazhong Agricultural University, Wuhan, China; ^2^School of Environmental Sciences, University of Guelph, Guelph, ON, Canada

**Keywords:** *Bacillus amyloliquefaciens*, *Bacillus velezensism*, biocontrol, *Plasmodiophora brassicae*, rapeseed

## Abstract

Clubroot disease caused by *Plasmodiophora brassicae* can lead to serious yield losses in crucifers such as *Brassica napus*. In this study, 323 bacterial strains were isolated from the rhizosphere of severely diseased *B. napus* in Dangyang county, Hubei province, China. Antagonistic strains were first identified based on dual culture inhibition zones with *Fusarium oxysporum* and *Magnaporthe oryzae*. These were then further screened in germination inhibition and viability assays of resting spores of *P. brassicae*. Finally, eight of the antagonistic strains were found to significantly reduce the disease severity of clubroot by more than 40% under greenhouse conditions, and two strains, F85 and T113, were found to have efficacy of more than 80%. Root hair infection experiments showed that F85 and T113 can inhibit early infection of root hairs, reduce the differentiation of primary plasmodia of *P. brassicae*, and inhibit formation of secondary zoosporangia. Based on sequence analysis of 16S rDNA gene, *gyrA* gene and 22 housekeeping genes as well as carbon source utilization analysis, the F85 was identified as *Bacillus velezensis* and T113 as *Bacillus amyloliquefaciens*. Genome analysis, PCR and RT-PCR detection revealed that both F85 and T113 harbor various antibiotic biosynthesis gene clusters required to form peptides with antimicrobial activity. To our knowledge, this is the first report of *B. velezensis* as a biocontrol agent against clubroot disease.

## Introduction

Clubroot, caused by soil-borne obligate parasite *Plasmodiophora brassicae*, is a serious disease on *Brassica* spp., especially oil-seed crops, and causes severe yield losses worldwide (Dixon and Page, 1998). Recently, clubroot disease has been increasing annually in Chongqing, Sichuan, Yunnan, and Hubei provinces and other major rapeseed-producing areas in China (Huang, 2010), and become the main disease on rapeseed. After rapeseed roots become infected, root cells proliferate abnormally, forming tumor-like bulges. The enlarged roots become cracked and then rotten due to infection by other pathogens and saprophytes. Life cycle of the pathogen consists of two key phases: in the initial phase, resting spores penetrate root hairs and epidermal cells and then form primary plasmodia; in the second phase, primary plasmodia release secondary zoospores, which then penetrate the root cortex and form galls. The mature secondary plasmodia of root cortex can form many resting spores that are released into soil (Schwelm et al., 2015).

Biocontrol with microbial antagonists has emerged as a promising alternative treatment with low environmental impact to reduce the use of synthetic fungicides. In recent decades, some antagonistic microorganisms have been identified as biological control agents (BCAs) for clubroot disease, including *Trichoderma* spp., *Bacillus subtilis*, *Bacillus Amyloliquefaciens*, and *Lysobacter antibioticus* (Cheah et al., 2000; Li et al., 2013; Zhou et al., 2014). *Trichoderma* can effectively prevent clubroot disease after mixing into an organic fertilizer containing actinomycetes (Joo et al., 2004). Soil-drench application of *L. antibioticus* cell-free culture filtrate reduced clubroot by 74.6% in greenhouse experiments (Zhou et al., 2014). The strain *B. subtilis* XF-1 exhibits a good suppression effect on *P. brassicae* with 76.9% control efficiency, and produces eight homologs of fengycin, seven homologs of dehydroxyfengycin, and six unknown fengycin-type cyclopeptides to destroy the cell wall integrity of the resting spores (Li et al., 2012).

Previous studies indicate that *Bacillus* spp. could produce many secondary metabolites to inhibit pathogen infection in host (Kilian et al., 2000). Lipopeptide antibiotics are a class of major antagonists produced by *Bacillus*. Based on chemical structure, lipopeptides have been divided into three major families: iturin, surfactin, and fengycin (Bonmatin et al., 2003). Lipopeptides are also involved in induction of resistance in plant systems (Ongena et al., 2007). Bacilysin, which is produced and excreted by many *Bacillus*, is a dipeptide antibiotic that resists pathogens, *Staphylococcus aureus* and *Candida albicans* (Yazgan et al., 2001). Flagellin produced by *Bacillus* spp. is a potent elicitor of defense responses in tomato and *Arabidopsis* (Felix et al., 1999). *Bacillus* spp. induces systemic resistance (ISR) of plants against a broad spectrum of phytopathogens (Ryu et al., 2004). For example, *B. cereus* AR156 induces ISR by salicylic acid/ethylene-signaling pathways in an NPR1-dependent manner which involves multiple PAMP-triggered immunity (PTI) components (Nie et al., 2017). *Bacillus amyloliquefaciens* SQR9 produces multiple elicitors to induce systemic resistance in *Arabidopsis* against DC3000 and *Botrytis cinerea* (Wu et al., 2018).

These lipopeptides, polyketides, dipepetide antibiotics, 2,3-butanediol, and exopolysaccharides play major roles in ISR (Wu et al., 2018). *Bacillus* strains have also been investigated for their capacity to protect plants by stimulating plant growth and forming multicellular structures or biofilms (Gardener, 2010; Chen et al., 2013). Rhizosphere bacteria were frequently found to form biofilm-like structures on plant roots (Morris and Monier, 2003), and biofilms from rhizobacteria play an important role in protecting plants (Timmusk et al., 2005). For example, GltB regulates biofilm formation and influences colonization of *B. subtilis* 916 on rice stems. Loss of *gltb* leads to poor efficacy against rice sheath blight (Zhou et al., 2016). Biocontrol effects of *Bacillus* on *P. brassicae* mainly include antagonistic activity (Li et al., 2013), induction of host resistance (Lahlali et al., 2013) and changes in microbial communities in the rhizosphere soil (Liu et al., 2018). However, there has been no report about biocontrol by *Bacillus velezensis* of clubroot.

The objectives of this study were to: (i) isolate effective antagonistic strains from the rhizosphere soil of asymptomatic rapeseed in severely infected fields; (ii) identify and characterize the selected bacterial isolates with antagonistic activities against *P. brassicae*; (iii) evaluate the efficacy of strains under greenhouse conditions; and (iv) identify main antimicrobial genes involved in antifungal activity in genomes of the *Bacillus* isolates. The goal was to identify new promising candidates to be used as BCAs against clubroot.

## Materials and Methods

### Soil Sample, Pathogen Inoculum, and Plant

From 2016 to 2017, soils from 5 to 15 cm deep associated with asymptomatic rapeseed were collected from severely infected fields in Dangyang county, Hubei province, China for isolation of antagonistic strains to *P. brassicae* and stored at 4°C. Clubroot galls were collected from infested rapeseed, dried at room temperature and stored at −20°C until used. Based on the method of Castlebury et al. (1994), crude resting spores were extracted. Approximately, 5 g of dried galls were soaked in 50 ml sterile deionized water (SDW) and then macerated in a blender at high speed for 2 min. The resulting suspension was filtered through eight layers of cheesecloth. Concentrations of resting spores were estimated with a hemocytometer and spore suspensions were diluted with SDW to 1 × 10^7^ spores ml^–1^ for soil inoculation.

For measuring germination inhibition and viability assay of the resting spores of *P. brassicae*, the crude resting spore suspension described above was purified. It was first centrifuged at 500 RPM for 10 min. The supernatant was then centrifuged at 3100 RPM for 15 min. Pellet was re-suspended in 30 ml of SDW and centrifuged for three times. The pellet was suspended again in 5 ml of 50% sucrose and centrifuged again. Then, supernatant was centrifuged under the same conditions again. Finally, the pellet was re-suspended in 5 ml of SDW and the purified suspension was stored in a refrigerator at 4°C for 1 week.

The susceptible oilseed rape cv. Huabu-9 was used for *P. brassicae* inoculation in greenhouse experiments. Seeds were sterilized in 1% sodium hypochlorite for 5 min and washed three times with sterile water. The disinfected seeds were placed into Petri dishes on moist filter paper at 25°C for germination. After 4 days, seedlings were transplanted into plastic pots (10 cm × 10 cm × 15 cm) filled with soil (five seedlings per pot). On the 10^th^ day after transplanting, the plants were inoculated with *P. brassicae*.

### Isolation and Screening of Antagonistic Strains Against *P. brassicae*

Approximately 10 g of rhizosphere soil was placed in 100 ml SDW, and then shaken for 30 min at 180 RPM. Suspensions were then diluted with SDW to 10^–3^, 10^–4^, 10^–5^ and 10^–6^, and 200 μl of each diluent was plated on Martin Medium (Wang et al., 2011). The plates were incubated at 28°C and colony morphology was observed every day. The isolates were purified on Martin Medium and stored at 4°C (Lai et al., 2012).

Since cell walls of *P. brassicae* are similar to that of *Fusarium oxysporum* and *Magnaporthe oryzae* (Moxham and Buczacki, 1983; Liu Y. et al., 2014), these two phytopathogens (*F. oxysporum* strain F685 and *M. oryzae* strain P131) were chosen as indicator fungi to screen antagonistic strains against *P. brassicae*. A hyphal plug of the pathogen was placed in center of each fresh 9-cm-diam PDA plates. A colony from a freshly prepared bacterial culture was streaked around fungal plug at a distance of 3 cm using a sterile toothpick. Culture media streaked around fungal plug served as a control. Each treatment was repeated on four plates. The plates were incubated at 28°C and inhibition zones were measured after 3 days.

### Germination Inhibition and Viability Assay of Resting Spores of *P. brassicae*

Root exudates stimulate germination of resting spores of *P. brassicae* (Rashid et al., 2013). Five-day-old rapeseed seedlings were transplanted individually onto a 50-ml beaker containing Hoagland’s nutrient solution and incubated at 24°C for 7 days. Root exudates were collected, filtered through a 0.22-μm cellulose nitrate filter and stored at 4°C until use. Single colonies of bacterial antagonistic strains were incubated on Luria-Bertani (LB) liquid medium at 30°C for 3 days at 180 RPM to prepare bacterial fermentation. The fermentation filtrate of bacterial isolates was centrifuged at 8000 RPM for 15 min and the supernatant was filtered through 0.22-μm cellulose nitrate filter.

Shallow liquid cultures in 50 ml flasks were used for germination inhibition assays of resting spores (Lahlali and Peng, 2014). Resting spore suspensions were diluted to 1 × 10^7^ spores ml^–1^ with root exudates and pH was adjusted to 6.3 with 2M HCl. Then, 4.5 ml resting spore suspension and 0.5 ml bacterial fermentation filtrate were placed in the 50 ml flask. As a control, resting spore suspension with LB medium was incubated at the same time. All treatments were allowed to incubate under dark conditions at 25°C. After 7 days, 10 μl of suspension was placed onto a glass slide. After slight heat drying over an alcohol lamp, 50 μl of 1% orcein was added to each slide and allowed to stain for 2–3 min, and then rinsed with 95% alcohol. After drying, 10% sterilized glycerol was added and the resting spores were observed under at BS203 optical microscope. Uncolored resting spores were considered germinated (Lahlali et al., 2011). Approximately, 200 spores were examined for each treatment, with three replications. The corrected germination rate of resting spores was equal to the spore germination rate after treatment minus the rate before treatment. Germination inhibition rates of resting spores were calculated using the formula n = [(a-b)/a] ×100, where n is germination inhibition rate of resting spores, a is resting spore germination rate of the control, and b is that of the treatment.

To test viability, 0.5 ml of a suspension of resting spores (4 × 10^7^ spores ml^–1^) was mixed with 0.5 ml of bacterial fermentation filtrate in a sterile tube, and incubated at room temperature. After 2 days, each treatment was mixed with an equal volume of phosphate buffer at pH 7.4 and added to 1 ml of Evans blue solution (vital stain). After mixing, it was allowed to stand for 30 min at room temperature, washed four times with distilled water, and resting spores were observed at BS203 microscope. Resting spores with dark blue color were considered as inactive. Sterile LB medium was used as control. Each experiment was repeated three times. At least 200 spores were examined from each treatment, with three repetitions. The corrected frequency for non-viable resting spores was equal to frequency of non-viable spores after treatment minus that before treatment.

### Greenhouse Experiments

In order to screen effective biocontrol strains against clubroot, soil-drench application of bacterial fermentations of all the antagonistic strains previously selected was done in the greenhouse. Each pot (10 cm × 10 cm) was inoculated with 10 ml of each bacterial fermentation described above, and each pot was inoculated with 10 ml resting spore suspension at the same time. Pots were treated with 20 ml water as the negative control and the fungicide Fluazinam was used as a positive control. Three pots were used for each treatment with three independent replicates. The treated plants were kept in a growth chamber at 25°C (16-h photoperiod, 90% RH) for 4 weeks. Clubroot severity was rated using a slightly improved grading standard (Peng et al., 2011) which included 0–3 scales: 0 = normal root growth without galling, 1 = galls on main roots or a few small galls formed on <1/3 lateral roots, 2 = galling on main root or on 1/3–2/3 lateral roots, and 3 = larger galls were formed on 2/3 of main root and lateral roots. Disease severity index (DSI) was calculated following Lahlali et al. (2013) and based on the grade standard.

### Effect of Antagonistic Strains on Early Infection of *P. brassicae* in Root Hairs

Sandy soil was used to cultivate seedlings for observation of root hair infection. A 50-ml beaker was filled with washed and sterilized sandy soil, and wrapped with black paper to create a dark environment for germination and infestation by resting spores. Three 7-day-old rapeseed seedlings were transplanted into each beaker. Three days after transplanting, the seedlings were inoculated with 1 ml of fermentation filtrate of biocontrol strain F85 or T113 and 1 × 10^7^ resting spores. The control was resting spore suspension alone. Each treatment was repeated three times. After inoculation, root hairs were monitored for 15 days, with nine seedlings observed for each treatment every day. Roots were washed with water, immersed in fixative solution (1:1; 95% acetic acid: 95% ethanol) for 10 min, stained in 125 ppm aniline-blue (dissolved in 50% acetic acid) for 1 min, and then rinsed with tap water for 1 min (Sharma et al., 2011). Root hairs of 1 cm in length of lower sections of hypocotyls was microscopically observed for infection.

### Identification and Characterization of T113 and F85

Morphological and physiological properties and molecular characteristics of isolates F85 and T113, which were identified as having high biocontrol efficacy in greenhouse experiments were tested. To observe colony morphology, a LB medium plate was streaked and incubated at 30°C for 24 h. Gram reaction was determined by using bioMe’rieux Gram stain kit (Sooyeon et al., 2014) according to the manufacturer’s instructions. The two strains were tested for their utilization patterns of carbon sources using the BIOLOG GEN III system (El-Liethy et al., 2018) based on the manufactures’ protocols. MicroPlates were read automatically by BIOLOG GEN III system and supplied database was used to characterize the strains.

Bacterial samples were first identified based on analysis of their 16S rDNA sequence. Genomic DNA of the strains was extracted using Bacterial Genomic DNA Extraction kit (TIANamp). The 16S rDNA was amplified using universal primers 27F (5′-AGAGTTTGATCCTGGCTCAG-3′) and 1492R (5′-GGTTACCTTGTTACGACTT-3′). Each PCR reaction was carried out in a final volume of 25 μl, containing 12.5 μl 2× PCR Mix, 1 μl of each primer, 1 μl genomic DNA and 9.5 μl ddH_2_O. The sample was subjected to the following temperature cycling profile: 94°C for 5 min, followed by 35 cycles of 94°C for 30 s, 50°C for 30 s, 72°C for 1.5 min, with a final extension step of 10 min at 72°C. PCR products were detected by 1% agarose gel electrophoresis and sequenced by the TSINGKE Biological Technology Company (Beijing, China). The 16S rDNA sequences were compared with GenBank databases^1^ using the BLAST search program. Second, to confirm the BLAST results, *gyrA* sequences were amplified with primers *gyrA*-f (5′-CAGTCAGGAAATGCGTACGTCCTT-3′) and *gyrA*-r (5′-CAAGGTAATGCTCCAGGCATTGCT-3′) (Chen et al., 2016) to construct a phylogenetic tree. The determined *gyrA* sequences were compared with GenBank databases and aligned with CLUSTALX (Jeanmougin et al., 1998). MEGA 7.0 software (Tamura et al., 2007) was used to conduct phylogenetic analyses using Maximum-likelihood method with 1000 bootstrap tests.

To further confirm the species identities of T113 and F85, 22 housekeeping genes, including *dnaG, frr, infC, nusA, pgk, pyrG, rplA, rplB, rplC, rplD, rplE, rplF, rplK, rplL, rplP, rplT, rpmA, rpoB, rpsB, rpsM, smpB*, and *tsf*, were extracted from the assembled genomes of F85 and T113, and chosen to splice sequences for analyze the phylogenetic relationship in *Bacillus* species using Maximum-likelihood method as described above.

### Genomic Sequencing and Annotation

Genomic DNA of T113 and F85 was extracted using Wizard^®^ Genomic DNA Purification Kit (Promega) according to the manufacturer’s protocol. For Illumina sequencing, at least 1 μg genomic DNA was used for each strain in sequencing library construction. DNA samples were sheared into 400–500 bp fragments using a Covaris M220 Focused Acoustic Shearer following the manufacture’s protocol. Illumina sequencing libraries were prepared from sheared fragments using NEXTflex^TM^ Rapid DNA-Seq Kit. Prepared libraries were then used for paired-end Illumina sequencing (2 bp × 150 bp) on an Illumina HiSeq X Ten platform (Majorbio Bio-pharm Technology, Shanghai, China).

Data generated were used for bioinformatic analysis using I-Sanger Cloud Platform^2^ from Shanghai Majorbio, using procedures as follows. Original image data were transferred into sequence data via base calling, which were defined as raw data or raw reads and saved as FASTQ files. Those FASTQ files were the original data provided for users, which included detailed read sequences and read quality information. Statistical analysis of quality information was performed for quality trimming, which removed low quality data could be removed to leave a clean data. An assembly of clean reads was performed using SOAPdenovo2.

Glimmer v3.02^3^ was used for coding sequence (CDS) prediction, tRNA-scan-SE v2.0.5^4^ was used for tRNA prediction, and Barrnap^5^ was used for rRNA prediction (Guo et al., 2012; Dunlap et al., 2013). The predicted CDSs were annotated from NR, Swiss-Prot, Pfam, GO, COG, and KEGG database using sequence alignment tools such as BLAST, Diamond and HMMER. Briefly, each set of query proteins were aligned with the databases, and annotations of best-matched subjects (e-value < 10^–5^) were obtained for gene annotation.

### Identification and Expression of Antimicrobial Biosynthesis Genes

PCR detection was performed to detect antimicrobial peptide biosynthetic genes with pre-existing primers (Mora et al., 2011; Yang et al., 2015; Sajitha et al., 2016). Amplification of 15 antimicrobial biosynthesis genes was performed using specific primers (Table 1). PCR amplifications were conducted in 25 μL of reaction mixtures containing 12.5 μl 2× PCR Mix, 1 μl PCR primer, 1 μl genomic DNA and 9.5 μl ddH_2_O. The sample was subjected to the following temperature cycling profile: 94°C for 5 min; followed by 35 cycles of 94°C for 30 s, 50°C for 30 s, 72°C for 1.5 min, with a final extension of 10 min at 72°C. PCR products were detected using 1% agarose gel electrophoresis.

**TABLE 1:** Specific primers for genes encoding antifungal peptides.

**Peptide**	**Gene**	**Primer name**	**Primers (5′–3′)**	**Tm (**°**C)**	**bp**
Surfactin	*srfAA*	srfAA-F	TCGGGACAGGAAGACATCAT	60	201
		srfAA-R	CCACTCAAACGGATAATCCTGA		
Fengycin	*fenB*	fenB-F	CTATAGTTTGTTGACGGCTC	55	1400
		fenB-R	CAGCACTGGTTCTTGTCGCA		
	*fenD*	fenD-F	GGCCCGTTCTCTAAATCCAT	60	269
		fenD-R	GTCATGCTGACGAGAGCAAA		
Iturin	*ITUDI*	ITUDI-F	GATGCGATCTCCTTGGATGT	59	650
		ITUDI-R	ATCGTCATGTGCTGCTTGAG		
	*ituD*	ituD-F	TTGAAYGTCAGYGCSCCTTT	58	482
		ituD-R	TGCGMAAATAATGGSGTCGT		
	*ituA*	ituA-F	ATGAAAATTTACGGAGTATATATG	53	1150
		ituA-R	TTATAACAGCTCTTCATACGTT		
	ituC	ituC-F	GGCTGCTGCAGATGCTTTAT	60	423
		ituC-R	TCGCAGATAATCGCAGTGAG		
Mycosubtilin	*mycB*	mycB-F	ATGTCGGTGTTTAAAAATCAAGTAACG	60	2024
		mycB-R	TTAGGACGCCAGCAGTTCTTCTATTGA		
Bacillomycin	*bmyB*	bmyB-F	GAATCCCGTTGTTCTCCAAA	60	370
		bmyB-R	GCGGGTATTGAATGCTTGTT		
	*bmyD*	bmyD-F	TTGAAYGTCAGYGCSCCTTT	51	482
		bmyD-R	TGCGMAAATAATGGSGTCGT		
Bacilysin	*BACD*	BACD-F	AAAAACAGTATTGGTYATCGCTGA	52	749
		BACD-R	CCATGATGCCTTCKATRCTGAT		
	*BACAB*	BACAB-F	CTTCTCCAAGGGGTGAACAG	61	815
		BACAB-R	TGTAGGTTTCACCGGCTTTC		
	*BAC*	BAC-F	CAGCTCATGGGAATGCTTTT	60	498
		BAC-R	CTCGGTCCTGAAGGGACAAG		
Flagellin	*hag*	hag-F	ATGAGAATCAACCACAATATCGC	54	1210
		hag-R	TTAACCTTTAAGCAATTGAAGAA		
Antimicrobial component	*tasA*	tasA-F	ATGGGTATGAAAAAGAAATTAAG	52	786
		tasA-R	TTAGTTTTTATCCTCACTGTGA		

Expression of antimicrobial biosynthesis genes was analyzed using reverse transcription PCR (RT-PCR). Samples were collected from bacteria grown for 1 day in liquid LB. Total RNA was extracted with the OMEGA Bio-tek Bacterial RNA Kit (OMEGA, Guangzhou, China). Reverse transcription assays were performed using TransScriptTM One-Step Gdna Removal and cDNA Synthesis SuperMix Kit (TransGen, Beijing, China). RT-PCR mixtures were composed of 12.5 μL of 2× HieffTM PCR Master Mix (YeSen, Shanghai, China), 1 μL of cDNA, 1 μL of each primer, and 9.5 μL nuclease-free water. The experiment was repeated three times.

To further determine whether antimicrobial biosynthesis genes were expressed upon interaction with *P. brassicae*, resting spores were treated with F85 or T113 bacterial cells for germination inhibition assays as described above. Total RNA was extracted from suspensions at 0, 12, 14, and 16 h after treatment. The F85 or T113 bacterial suspension treated with root exudates served as a control. RT-PCR manipulation was conducted as described above.

### Statistical Analysis

Data were subjected to analyses of variance (ANOVA) using SPSS 13.0 software (SPSS, Inc., Chicago, IL, United States). Mean comparisons were conducted using a least significant difference (LSD) test (*P* = 0.05).

## Results

### Screening of Antagonistic Bacterial Strains Against *P. brassicae*

**FIGURE 1:**
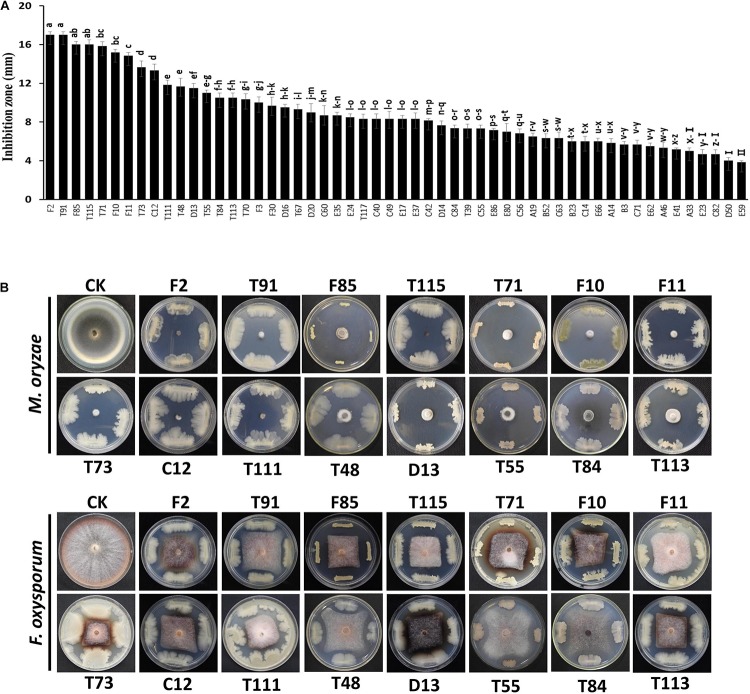
Streak plate method to screen antagonistic strains by using indicator fungi, *Fusarium oxysporum* and *Magnaporthe oryzae*. **(A)** Average size of inhibition zones of the two indicator fungi. Each experiment was repeated three times. **(B)** Inhibition zones of biocontrol strains against the two fungi. The red line indicated the inhibition zones greater than 10 mm as the cut-off value. Data are presented as mean ± SD. Different letters indicate significant differences between treatments according to LSD test (*P* < 0.05). Capped lines represent standard errors.

A total of 323 bacterial strains were isolated from the rhizosphere of asymptomatic oilseed rape plants in severely diseased fields. Among these bacterial strains, 54 were found to have potential antagonistic activities in dual culture streak plates, as their average inhibition of the two indicator fungi was greater than 3 mm (Figures 1A,B). Notably, 17 strains (F2, T91, F85, T115, T71, F10, F11, T73, C12, T111, T48, D13, T55, T84, T113, T70, and F3) had inhibition zones greater than 10 mm (Figure 1A). These 54 bacterial strains with potential antagonistic activities were selected for further testing.

### Inhibitory Effects of Antagonistic Strains on Germination and Viability of Resting Spores

**FIGURE 2:**
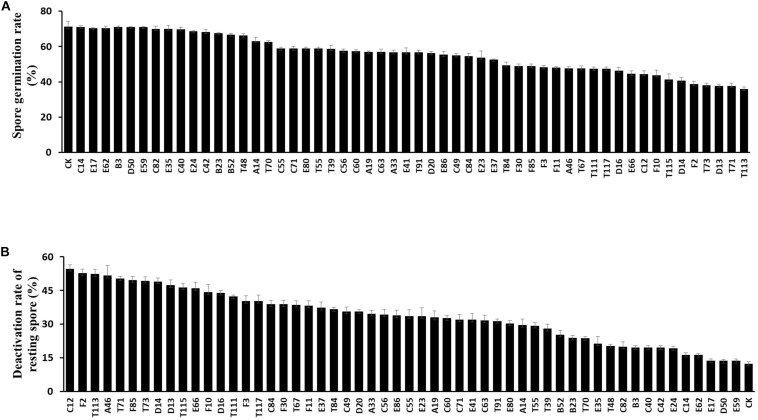
Inhibitory effect of antagonistic strains on the germination and viability of resting spores. **(A)** Resting spore germination rate of *Plasmodiophora brassicae* after treatment with biocontrol strains. Each experiment was repeated three times. Data are presented as mean ± SD. Capped lines represent standard errors. **(B)** Deactivation rate of resting spores of *P. brassicae*.

The 54 bacterial strains with inhibition zone greater than 3 mm were tested for their inhibitory effects on germination and viability of resting spores. After staining with orcein dye, 20 strains, including T84, F30, F85, F3, F11, A46, T67, T111, T117, D16, E66, C12, F10, T115, D14, F2, T73, D13, T71 and T113, were found to have large inhibitory effects on the germination rate of resting spores (36–49 vs. 71% in the control) (Figure 2A). Particularly, strain T113 produced the lowest resting spore germination rate at 36%.

All 54 bacterial strains were also tested for their inhibitory effects on viability of resting spores based on vital staining with Evan’s blue. Thirteen strains, C12, F2, T113, A46, T91, F85, T73, D14, D13, T115, E66, F10 and D16, showed high deactivation effects on resting spores (45–55 vs. 12% in the control) (Figure 2B), particularly C12, which resulted in a maximum deactivation rate (54.7%) of resting spores.

### Biocontrol Effects of Antagonistic Strains on Clubroot Disease Under Greenhouse Conditions

**FIGURE 3:**
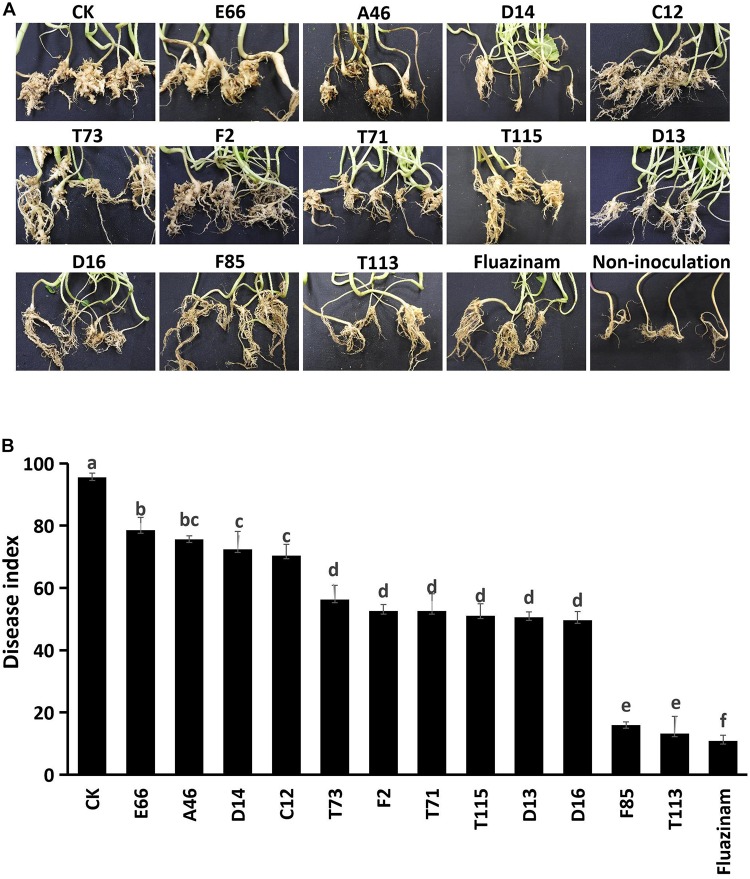
Control efficiency of the biocontrol bacterial strains against *P. brassicae* in greenhouse. **(A)** Morphology of rapeseed roots of each bacterial treatment. **(B)** Disease incidence of clubroot treated with bacterial suspensions. Each experiment was repeated three times. Different letters indicate significant differences between different treatments according to LSD test (*P* < 0.05). Capped lines represent standard errors.

To investigate the suppressive effects of antagonistic bacteria on clubroot, strains E66, A46, D14, C12, T73, F2, T71, T115, D13, D16, F85, and T113 with potent inhibitory effects on germination and viability of resting spores were tested under greenhouse conditions. The positive control, fungicide Fluazinam, effectively suppressed clubroot resulting in a disease index of 10.9. Disease index of rapeseeds inoculated with *P. brassicae* alone was 95.6, whereas treatment with different antagonistic bacterial strains reduced the disease index to 10–80. Among these strains, F85 showed significant suppression activities on *P. brassicae* with a disease index value of 15.9 and T113 was 13.2 (Figure 3).

### Effect of Antagonistic Strains on Early Infection of *P. brassicae*

**FIGURE 4:**
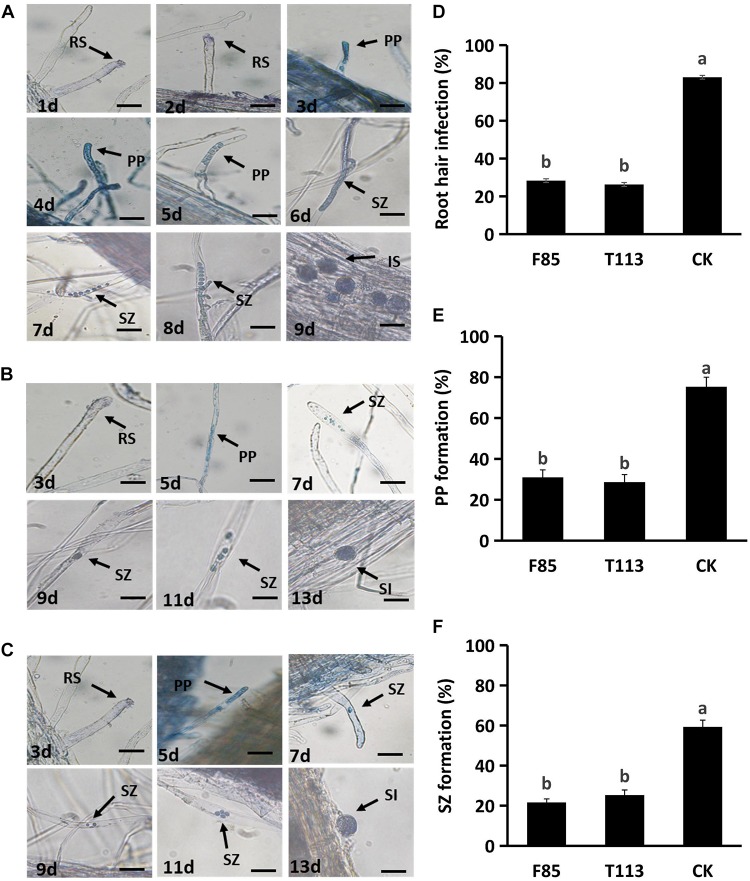
Inhibition of the infection process of *P. brassicae* on rapeseed root hairs by F85 and T113. **(A)** Root hair infection process of untreated control. The zoospores infected root hairs in 2 DAI, formed primary plasmodia in 3 DAI, secondary zoosporangia in 6 DAI and started secondary infection in 9 DAI. RS, resting spore; PP, primary plasmodia; SZ, secondary zoosporangia; SI, secondary infection. **(B)** Root hair infection process of T113 treatment. The zoospores infected root hairs in 3 DAI, formed primary plasmodia in 5 DAI, secondary zoosporangia in 7 DAI, and started secondary infection in 15 DAI. **(C)** Root hair infection process of F85 treatment. The zoospores infected root hairs in 3 DAI, formed primary plasmodia in 5 DAI, secondary zoosporangia in 7 DAI and started secondary infection in 15 DAI. **(D)** Percentage of root hair infection (%) after treated with F85 or T113. **(E)** Percentage of primary plasmodium formation (%). **(F)** Percentage of second zoosporangia formation (%). CK: water. Different letters indicate significant differences between treatments according to LSD test (*P* < 0.05). Capped lines represent standard errors.

Strains F85 and T113, which produced the best disease suppression effect, were selected for root hair infection experiments. In blank control treatment, zoospores infected the root hairs at 2 days after inoculation (DAI) and primary plasmodia were formed at 3 DAI. Secondary zoosporangia developed in root hairs at 6 DAI and secondary zoospores infected the root cortex to result in secondary infection at 9 DAI (Figure 4A). Inoculation with F85 or T113, delayed and suppressed zoospore infection. Primary plasmodia were formed at 5 DAI and secondary zoosporangia in root hairs were observed at 7 DAI. Secondary zoospores infected cortex of root hairs to cause secondary infection at 13 DAI (Figures 4B,C). Compared with the control, the F85 or T113 treatment effectively inhibited root hair infection as well as formation of primary plasmodia and secondary zoosporangia (Figures 4D–F). Hence, both F85 and T113 could effectively suppress root hair infection of *P. brassicae*.

### Identification and Molecular Characterization of F85 and T113

**FIGURE 5:**
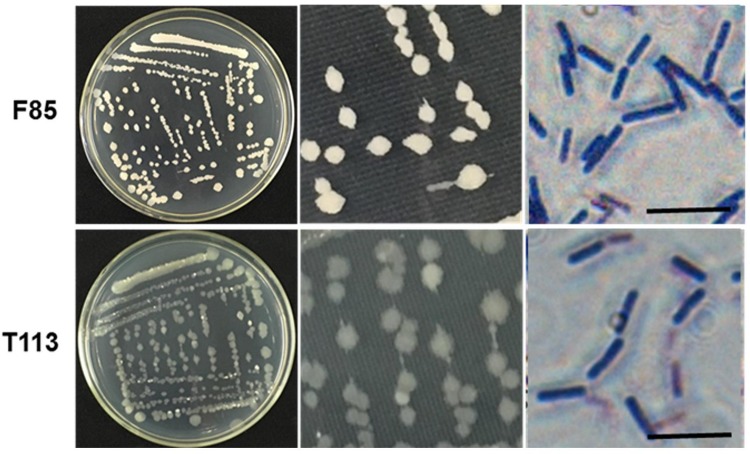
Colony morphology and gram staining of the F85 and T113. Both strains strained purpled, identifying them as Gram-positive bacteria. The cells were rod-shaped. The F85 colonies were khaki, the edges were clearcut, and the surface was dry and opaque. The colony of T113 had a dull color and a smooth, translucent surface.

Analysis of morphological, biochemical, physiological, and molecular characteristics of F85 and T113 were used for taxonomic identification. Both strains were found to be Gram-positive, rod-shaped bacteria (Figure 5). Characteristics detected by BIOLOG GEN III MicroPlate are shown in Supplementary Table S1. Based on these characteristics, F85 and T113 were automatically classified as *Bacillus* spp. which was also confirmed by sequence analysis of 16S rDNA gene.

Analysis of a partial *gyrA* sequence demonstrated that F85 was matched *B. velezensis* SCGB 1CP023320.1 at 99%, while T113 was matched *B. amyloliquefaciens* UCMB5036 at 99%.

**FIGURE 6:**
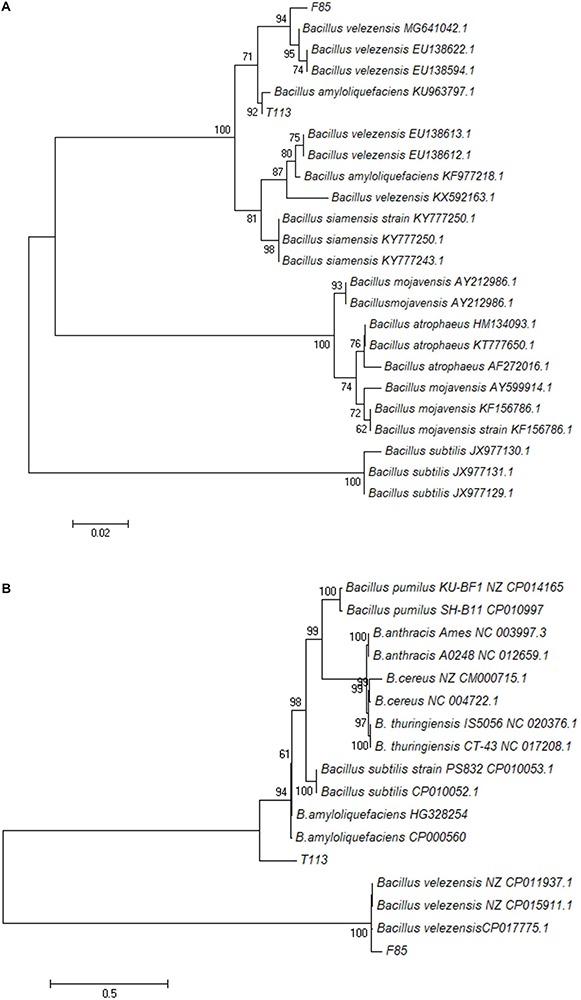
Phylogenetic trees based on the partial nucleotide sequence of *gyrA* **(A)** and 22 housekeeping genes **(B)**. A Maximum-likelihood phylogenetic tree of F85 and T113 was constructed using MEGA 7.0. The numbers at nodes indicate levels of bootstrap support (%) based on a Maximum-likelihood analysis of 1000 re-sampled datasets; only values greater than 50% are provided.

Sequence analysis of *gyrA* was carried out to assess its taxonomic position in the phylogenetic tree. The results indicated that F85 and *B. velezensis* were clustered in one group while T113 and *B. amyloliquefaciens* were clustered in another group, which were distinctly separated from other species of *Bacillus* (Figure 6A). To confirm the identification, we also constructed a phylogenetic tree based on 22 housekeeping genes from bacterial genomes. The phylogenetic analysis was consistent with previous results (Figure 6B).

Based on morphological, physiological and biochemical characteristics and the results of sequence analysis, F85 was identified as *B. velezensis* and T113 as *B. amyloliquefaciens*.

### Genomic Features and Antibiotic Biosynthesis Analysis of *B. velezensis* F85 and *B. amyloliquefaciens* T113

**FIGURE 7:**
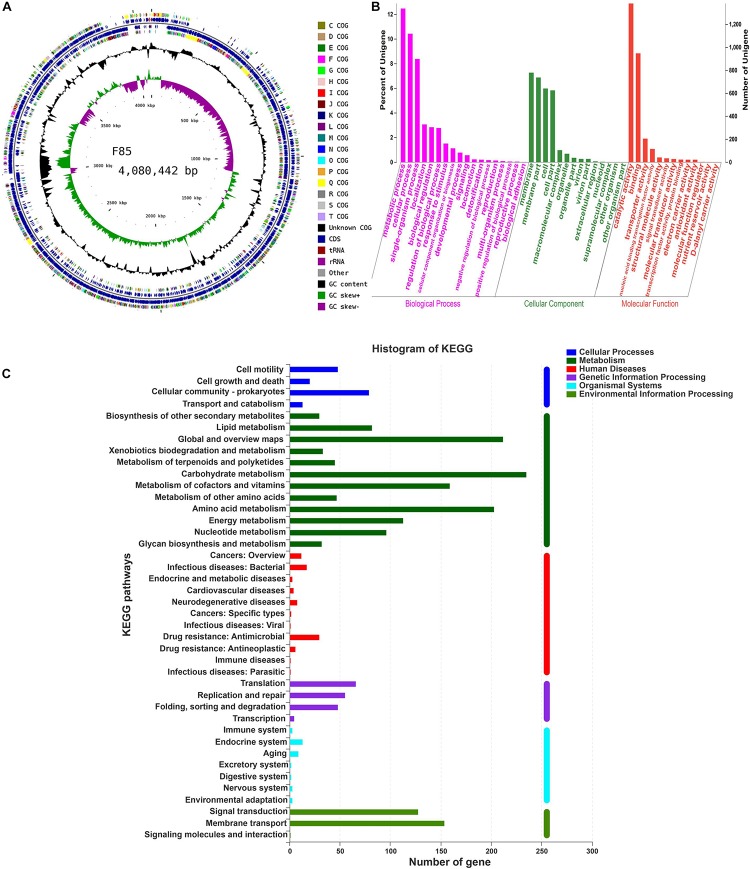
Circular genome visualization, and GO annotation and KEGG pathway analysis of F85. **(A)** Circular representation of the F85 genome. From outside to inside, circle 1: scale; circles 2 and 3: predicted CDSs color-coded according to their functions; circle 4: rRNA and tRNA; circle 5: GC content; circle 6: GC skew (G+C/G-C). **(B)** GO annotation of F85, mainly including three major categories: ‘biological processes,’ ‘cellular component,’ and ‘molecular function.’ **(C)** KEGG pathway analysis of F85. KEGG pathway can be divided into seven categories: Metabolism, Genetic Information Processing, Environmental Information Processing, Cellular Processes, Organismal Systems, Human Diseases, and Drug Development.

**FIGURE 8:**
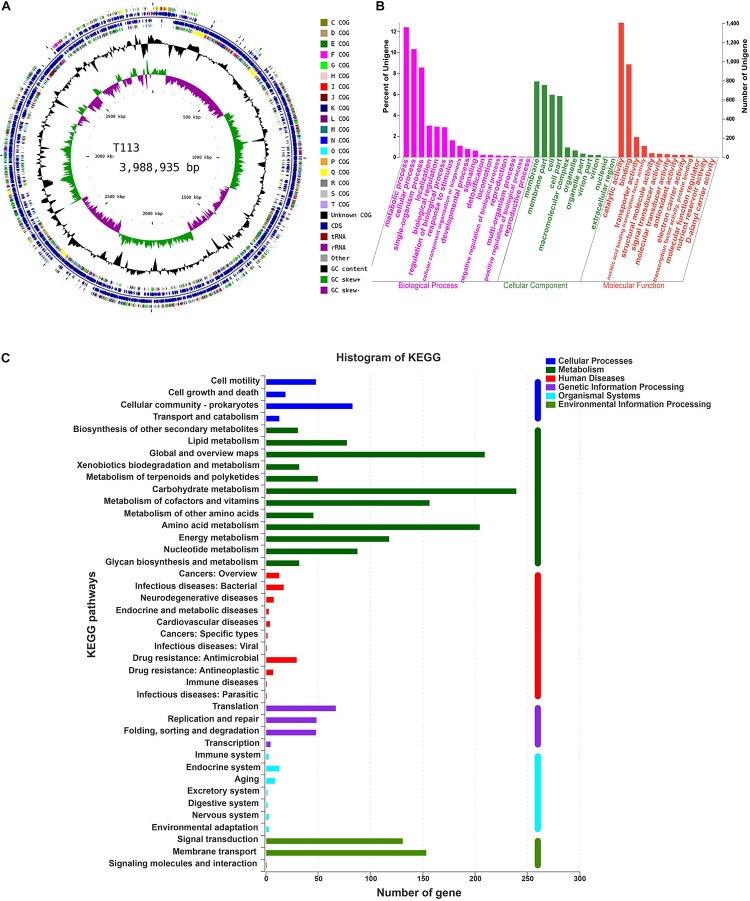
Circular genome visualization, and GO and KEGG pathway analysis of T113. **(A)** Circular representation of T113 genome. **(B)** GO annotation of T113. **(C)** KEGG pathway analysis of T113.

To investigate biosynthesis features of antibiotics in F85 and T113, two libraries were generated to investigate antibiotic biosynthesis genes. In general, both samples showed high quality, with >99% of base reads at Q20 (rate of bases with quality >20) and 96% of base reads >Q30 (rate of bases with quality >30) (Table 2). The distribution of base composition and quality on the clean reads are shown in Supplementary Figures S1, S2. The whole genomes of F85 and T113 were sequenced, revealing a complete circular genome 4,080,442 bp in length and a GC content of 45.98%, while the T113 genome was a complete circular genome 3,988,935 bp in length with a GC content of 46.31% (Table 3). The major features of F85 and T113 genomes are shown in Figures 7A, 8A as circular graphs.

**TABLE 2:** Summary of sequenced libraries for F85 and T113 after filtering and genome mapping.

**Summary**	**F85**	**T113**
Raw pair reads	4825305×2	5496122×2
Clean pair reads	4683052×2	5364342×2
Clean bases (bp)	1.4E+09	1.6E+09
Raw Q20 (%)	97.87	98.11
Raw Q30 (%)	94.7	95.14
Clean reads ratio (%)	96.03	96.50
Clean Q20 (%)	99.07	99.12
Clean Q30 (%)	96.71	96.86

**TABLE 3:** General features of the genome sequences of F85 and T113.

**Feature**	**F85**	**T113**
Genome size (bp)	4080442	3988935
GC content (%)	45.98	46.31
Scaffold number	41	81
CDS number	4258	4095
Repeat number	98	93
Gene number	4258	4095
Gene total length (bp)	3564948	3489816
Genes of KEGG	2181	2173
Genes of COG	3020	3000
tRNA number	51	61
rRNA number	2	4

Functional distributions of genes and their genetic relationships were assessed by Gene Ontology (GO) annotation and Kyoto Encyclopedia of Genes and Genomes (KEGG) pathway analysis. Annotation of F85 and T113 genomes revealed that main GO categories were ‘biological processes,’ ‘cellular component,’ and ‘molecular function’ (Figures 7B, 8B). A total of 2425 genes were annotated in the GO classification of F85 (Supplementary Table S2), and the most enriched terms for ‘biological processes’ were metabolic process (1338 genes), cellular process (1119 genes) and single-organism process (899 genes). The most enriched terms for the ‘cellular component’ were membrane (780 genes) and membrane part (738 genes). In the ‘molecular function,’ there were the largest numbers of genes in catalytic activity (1382 genes) and binding (948 genes). Almost 2500 genes were annotated in the GO classification of T113 (Supplementary Table S3). The most enriched terms for ‘biological processes’ were metabolic process (1361 genes), cellular process (1132 genes) and single-organism process (940 genes). The most enriched terms for the ‘cellular component’ were membrane (794 genes) and membrane part (757 genes). In the ‘molecular function,’ there were the largest numbers of genes in catalytic activity (1409 genes) and binding (973 genes).

A total of 2024 genes were annotated in the KEGG pathway of F85 (Supplementary Table S4). Representative pathways were ‘cellular processes’ (160 genes), ‘metabolism’ (1287 genes), ‘human diseases’ (85 genes), ‘genetic information processing’ (174 genes), ‘organismal systems’ (35 genes) and ‘environmental information processing’ (283 genes) (Figure 7C). For T113, 2027 genes were annotated in the KEGG pathway (Supplementary Table S2). The representative pathways were ‘cellular processes’ (163 genes), ‘metabolism’ (1287 genes), ‘human diseases’ (87 genes), ‘genetic information processing’ (169 genes), ‘organismal systems’ (35 genes) and ‘environmental information processing’ (286 genes) (Figure 8C).

Subsequently, antibiotic biosynthesis genes were detected from the enriched categories of GO annotation and KEGG pathway. Antibacterial metabolites could be grouped into two separate classes: ribosome-synthesized peptides such as bacteriocin, and small microbial peptides enzymatically synthesized by non-ribosomal pathways, mainly cyclic lipopeptides (Finking and Marahiel, 2004; Romero et al., 2007; Baruzzi et al., 2011). The F85 and T113 genomes contained antibiotic biosynthesis gene clusters associated with both ribosomal and non-ribosomal synthesized peptides (Tables 4, 5). In F85 and T113, these gene clusters comprised genes that encode non-ribosomal peptide synthetases mainly including lipopeptides, peptides, polyketide and terpenoids, and ribosomal peptide synthetases including bacteriocin and antibacterial proteins. The other secondary metabolites related to antibiotic synthesis were identified from the pathway database, including ansamycins, carbapenem, streptomycin, monobactam, phenylpropanoid, penicillin, and cephalosporin. The substances encoded by these genes might be involved in synthesis of these antibiotics.

**TABLE 4:** Predicted antibiotic biosynthesis genes within *Bacillus velezensis* F85 genome.

**Synthetase**				
**type**	**Compound**	**Locus**	**Genes**	**Annotation**
NRPS	Lipopeptides	gene4273	*ppsA*	Fengycin family lipopeptide synthetase A
		gene4274	*ppsB*	Fengycin family lipopeptide synthetase B
		gene4275	*ppsC*	Fengycin family lipopeptide synthetase C
		gene0001	*ppsD*	Fengycin family lipopeptide synthetase D
		gene0002	*ppsE*	Fengycin family lipopeptide synthetase E
		gene0029	*ituA*	Iturin family lipopeptide synthetase A
		gene0030	*ituB*	Iturin family lipopeptide synthetase B
		gene0031	*ituC*	Iturin family lipopeptide synthetase C
		gene1993	*srfAB*	Surfactin family lipopeptide synthetase B
		gene1994	*srfAC*	Surfactin family lipopeptide synthetase C
		gene4276	*srfAA*	Surfactin family lipopeptide synthetase A
	Peptide	gene1416	*bacA*	bacA; prephenate decarboxylase
		gene1417	*bacB*	3-[(4R)-4-Hydroxycyclohexa-1,5-dien-1-yl]-2-oxopropanoate isomerase
		gene1418	*bacC*	Dihydroanticapsin dehydrogenase
		gene1419	*bacD*	L-Alanine-L-anticapsin ligase
		gene1420	*bacE*	MFS transporter, DHA3 family, bacilysin exporter BacE
		gene1421	*bacF*	Bacilysin biosynthesis transaminase BacF
		gene1422	*bacG*	Bacilysin biosynthesis oxidoreductase BacG
	Polyketide	gene0153	*pksS*	Cytochrome P450 PksS
		gene0154	*pksR*	Polyketide synthase PksR
		gene0155	*pksN*	Polyketide synthase PksN
		gene0156	*pksM*	Polyketide synthase PksM
		gene0157	*pksL*	Polyketide synthase PksL
		gene0158	*pksJ*	Polyketide synthase PksJ
		gene0159	*pksI*	Polyketide biosynthesis enoyl-CoA hydratase PksI
		gene0160	*pksH*	Polyketide biosynthesis enoyl-CoA hydratase PksH
		gene0161	*pksG*	Polyketide biosynthesis 3-hydroxy-3-methylglutaryl-CoA synthase-like enzyme PksG
		gene0162	*acpK*	Polyketide biosynthesis acyl carrier protein
		gene0163	*pksE*	*Trans*-AT polyketide synthase, acyltransferase and oxidoreductase domains
		gene0164	*pksD*	Bacillaene synthase *trans-*acting acyltransferase
		gene0165	*pksC*	Polyketide biosynthesis malonyl-CoA-[acyl-carrier-protein] transacylase
	Terpenoid	gene0219	*dxr*	1-Deoxy-D-xylulose-5-phosphate reductoisomerase
		gene0221	*uppS*	Undecaprenyl diphosphate synthase
		gene0886	*atoB*	Acetyl-CoA *C-*acetyltransferase
		gene2645	*ispH*	4-Hydroxy-3-methylbut-2-en-1-yl diphosphate reductase
		gene2655	*gcpE*	(E)-4-Hydroxy-3-methylbut-2-enyl-diphosphate synthase
		gene2736	*dxs*	1-Deoxy-D-xylulose-5-phosphate synthase
		gene2752	*atoB*	Acetyl-CoA *C-*acetyltransferase
		gene2899	*idi*	Isopentenyl-diphosphate delta-isomerase
		gene2911	*hepST*	Heptaprenyl diphosphate synthase
		gene2913	*hepST*	Heptaprenyl diphosphate synthase
		gene4160	*ispF*	2-*C*-Methyl-D-erythritol 2,4-cyclodiphosphate synthase
		gene4161	*ispD*	2-*C*-Methyl-D-erythritol 4-phosphate cytidylyltransferase
		gene4209	*ispE*	4-Diphosphocytidyl-2-*C*-methyl-D-erythritol kinase
RPS	Bacteriocin	gene1826	*nisF*	Lantibiotic transport system ATP-binding protein
		gene1827	*nisE*	Lantibiotic transport system permease protein
		gene1828	*nisG*	Lantibiotic transport system permease protein
		gene1829	*nisR*	Two-component system, OmpR family, lantibiotic biosynthesis response regulator NisR/SpaR
	Antibacterial proteins	gene2701	*tasA*	Spore coat-associated protein N

**TABLE 5:** Predicted antibiotic biosynthesis genes within *Bacillus amyloliquefaciens* T113 genome.

**Synthetase**				
**type**	**Compound**	**Locus**	**Genes**	**Annotation**
NRPS	Lipopeptides	gene0001	*ppsC*	Fengycin family lipopeptide synthetase C
		gene0002	*ppsD*	Fengycin family lipopeptide synthetase D
		gene0003	*ppsA*	Fengycin family lipopeptide synthetase D
		gene0004	*ppsB*	Fengycin family lipopeptide synthetase A
		gene0006	*ppsE*	Fengycin family lipopeptide synthetase B
		gene0032	*ituB*	Iturin family lipopeptide synthetase B
		gene0033	*ituC*	Iturin family lipopeptide synthetase C
		gene0031	*ituA*	Iturin family lipopeptide synthetase A
		gene2096	*srfAB*	Surfactin family lipopeptide synthetase B
		gene2097	*srfAC*	Surfactin family lipopeptide synthetase C
		gene3964	*srfAA*	Surfactin family lipopeptide synthetase A
		gene3963	*srfAA*	Surfactin family lipopeptide synthetase A
	Peptide	gene1334	*bacG*	Bacilysin biosynthesis oxidoreductase BacG
		gene1335	*bacF*	Bacilysin biosynthesis transaminase BacF
		gene1336	*bacE*	MFS transporter, DHA3 family, bacilysin exporter BacE
		gene1337	*bacD*	L-Alanine-L-anticapsin ligase
		gene1338	*bacC*	Dihydroanticapsin dehydrogenase
		gene1339	*bacB*	3-[(4R)-4-Hydroxycyclohexa-1,5-dien-1-yl]-2-oxopropanoate isomerase
		gene1340	*bacA*	bacA; prephenate decarboxylase
	Polyketide	gene0156	*pksS*	Cytochrome P450 PksS
		gene0157	*pksR*	Polyketide synthase PksR
		gene0158	*pksN*	Polyketide synthase PksN
		gene0159	*pksM*	Polyketide synthase PksM
		gene0160	*pksL*	Polyketide synthase PksL
		gene0161	*pksJ*	Polyketide synthase PksJ
		gene0162	*pksI*	Polyketide biosynthesis enoyl-CoA hydratase PksI
		gene0163	*pksH*	Polyketide biosynthesis enoyl-CoA hydratase PksH
		gene0164	*pksG*	Polyketide biosynthesis 3-hydroxy-3-methylglutaryl-CoA synthase-like enzyme PksG
		gene0165	*acpK*	Polyketide biosynthesis acyl carrier protein
		gene0166	*pksE*	*Trans-*AT polyketide synthase, acyltransferase and oxidoreductase domains
		gene0167	*pksD*	Bacillaene synthase *trans-*acting acyltransferase
		Gene0168	*pksC*	Polyketide biosynthesis malonyl-CoA-[acyl-carrier-protein] transacylase
	Terpenoid	gene0224	*dxr*	1-Deoxy-D-xylulose-5-phosphate reductoisomerase
		gene0226	*uppS*	Undecaprenyl diphosphate synthase
		gene1018	*ispH*	4-Hydroxy-3-methylbut-2-en-1-yl diphosphate reductase
		gene1027	*gcpE*	(E)-4-Hydroxy-3-methylbut-2-enyl-diphosphate synthase
		gene1110	*dxs*	1-Deoxy-D-xylulose-5-phosphate synthase
		gene1128	*atoB*	Acetyl-CoA *C-*acetyltransferase
		gene1853	*atoB*	Acetyl-CoA *C-*acetyltransferase
		gene2469	*hepST*	Heptaprenyl diphosphate synthase
		gene2471	*hepST*	Heptaprenyl diphosphate synthase
		gene2483	*idi*	Isopentenyl-diphosphate Delta-isomerase
		gene3645	*ispF*	2-*C-*Methyl-D-erythritol 2,4-cyclodiphosphate synthase
		Gene3646	*ispD*	2-*C*-Methyl-D-erythritol 4-phosphate cytidylyltransferase
		gene3693	*ispE*	4-Diphosphocytidyl-2-*C*-methyl-D-erythritol kinase
RPS	Bacteriocin	gene2943	*nisF*	Lantibiotic transport system ATP-binding protein
		gene2944	*nisE*	Lantibiotic transport system permease protein
		gene2945	*nisG*	Lantibiotic transport system permease protein
		gene2946	*nisR*	Two-component system, OmpR family, lantibiotic biosynthesis response regulator NisR/SpaR
		gene2947	*nisK*	Two-component system, OmpR family, lantibiotic biosynthesis sensor histidine kinase NisK/SpaK
	Antibacterial proteins	gene1075	*tasA*	Spore coat-associated protein N

**FIGURE 9:**
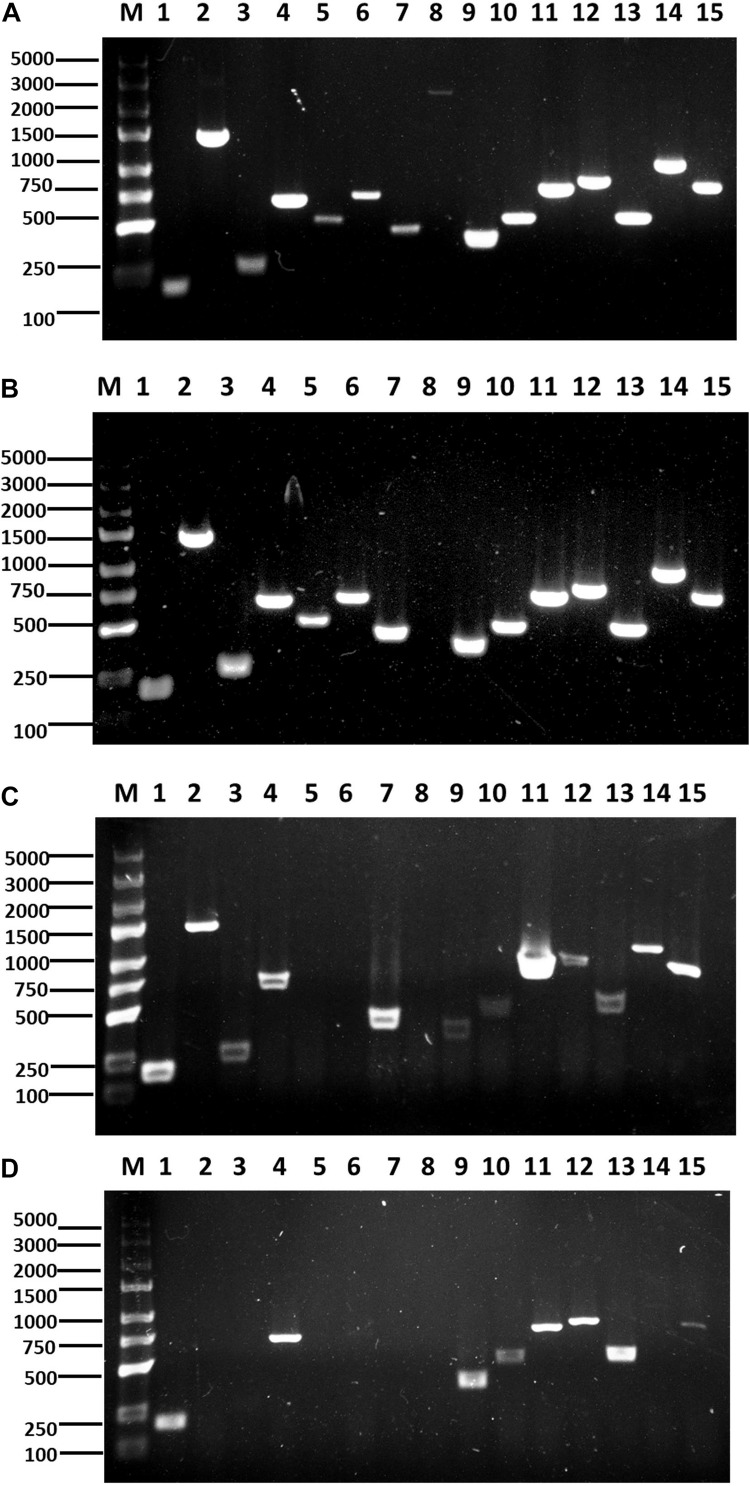
Identification and expression of antimicrobial biosynthesis genes. PCR amplification of lipopeptide biosynthetic genes from F85 **(A)** and T113 **(B)**. RT-PCR amplification of lipopeptide biosynthetic genes from F85 **(C)** and T113 **(D)**. Lane 1: *srfAA*; Lane 2: *fenB*; Lane 3: *fend*; Lane 4: *ITUDI*; Lane 5: *ituD*; Lane 6: *ituA*; Lane 7: *ituC*; Lane 8: *mycB*; Lane 9: *bmyB*; Lane 10: *bmyD*; Lane 11: *BACD*; Lane 12: *BACAB*; Lane 13: *BAC*; Lane 14: *hag*; Lane 15: *tasA*. Marker: TaKaRa DL5000 (TaKaRa, Dalian, China).

The antibiotic biosynthesis genes in *B. velezensis* F85 and *B. amyloliquefaciens* T113 were amplified to confirm presence of the genes in these genomes. PCR detection showed that all selected antibiotic biosynthesis genes were amplified in F85 and only *mycB* was not detected in T113 genomic DNA (Figures 9A,B). PCR products were finally confirmed by sequencing.

Expression levels of the 15 antibiotic biosynthesis genes in bacterial cells were further evaluated using RT-PCR, and the results showed that 12 genes (*srfAA, fenB, fend, ITUDI, ituC, mycB, bmyD, BACD, BACAB, BAC, hag* and *tasA*) were expressed in F85 while eight genes (*srfAA, ITUDI, bmyB, bmyD, BACD, BACAB, BAC*, and *tasA*) were expressed in T113 (Figures 9C,D). Then, other three non-expressed genes from F85 and seven non-expressed genes from T113 were all chosen to determine whether these antimicrobial biosynthesis genes were expressed upon interaction with *P. brassicae.* However, all selected genes were still not expressed. Hence, many antimicrobial biosynthesis genes were identified in F85 and T113, and expression of these genes could lead to the synthesis of antibiotic compounds.

## Discussion

In this study, isolation and screening of biocontrol bacteria from the rhizosphere of healthy plants in severely diseased fields were carried out. The assumption was that, if no symptoms had developed for plants growing in clubroot-infested soil, an antagonist may have prevented infection by *P. brassicae*. *P. brassicae* is an obligate parasite that cannot be cultured *in vitro*, which presents a bottleneck for the large-scale screening of biocontrol bacteria against *P. brassicae* (Bi et al., 2016). For screening of biocontrol bacteria against clubroot, a variety of pathogenic fungi could be used as indicator fungi for antagonistic test, particularly the pathogenic fungus *F. oxysporum*. The cell wall of *P. brassicae* is similar to that of *F. oxysporum* and *M. oryzae* (Moxham and Buczacki, 1983; Liu Y. et al., 2014). Fifty-four strains of biocontrol bacteria with apparent inhibition zones in dual plate cultures with the test pathogens were then screened in resting spore germination and viability assays, which are more precise and comprehensive than previous research methods. Two potential biocontrol strains, F85 and T113 both exhibited good inhibitory effects on germination and viability of resting spores (Supplementary Figure S3), and produced greater than 80% disease suppression in greenhouse experiments. The efficacy of these two strains is better than that of previously reported biocontrol strains (Jäschke et al., 2010; Lahlali et al., 2013; Zhou et al., 2014). In order to improve the efficiency of the biocontrol agents under high disease pressure, it will be necessary to examine the combined effect of F85 and T113 against *P. brassicae* in the future. BIOLOG analysis is always used to identify species of bacteria. However, it is really hard to distinguish characteristics of carbon source utilization between different species of *Bacillus.* In this case, the *gyrA* sequence provides a firm framework for the rapid and accurate classification and identification of *B. subtilis* and related taxa (Chun and Bae, 2000). In this study, F85 and T113 could be easily identified as *B. velezensis* and *B. amyloliquefaciens*, respectively, by the single gene *gyrA.* The results were consistent with identification using 22 housekeeping genes in phylogenetic analysis. Liu X. et al. (2014) reported that *B. amyloliquefaciens* strain HB-26 showed approximately 60% inhibition activity against *P. brassicae* in Chinese cabbage pot experiment (Liu X. et al., 2014). Noticeably, the *B. amyloliquefaciens* strain T113 used in this study exhibited higher suppressive effect on clubroot. Moreover, the present study reports the first description of the biological potential of F85, a *B. velezensis* strain, to reduce clubroot. However, further work is needed to explore mechanisms of these *Bacillus* strains to suppress infection of *P. brassicae*.

The lipopeptide antibiotics produced by *Bacillus* spp. have been generally assumed to have antifungal activity. Strains of *B. amyloliquefaciens* produce various antimicrobial lipopeptides including iturins, surfactins and fengycins against phytopathogens (Kim et al., 2010; Pathak and Keharia, 2014). *Bacillus subtilis* XF-1 is highly protective against *P. brassicae*. It was predicted to produce cyclic lipopeptide (CLP) antibiotics by genomic analysis, and the CLP were identified by LC/ESI-MS and LC/ESI-MS/MS as fengycin B, fengycin C, fengycin D, and fengycin S (Li et al., 2013). Most *Bacillus* species produce two to four antimicrobial peptides (AMPs) with bacteriostatic effects and structural stability (Mora et al., 2011). *Bacillus subtilis* YB-05 has at least four antifungal genes (*fenB*, *ituA*, *hag*, and *tas*) (Yang et al., 2015). *Bacillus subtilis* strain A1/3 showed exceptionally diverse antibiotic capacities compared with other *B. subtilis* strains. It has six AMP (antimicrobial peptides) genes, including *srf* (surfactin), *bacA* (bacylisin), *fenD* (fengycin), *bmyB* (bacyllomicin), *spaS* (bubtilin), and *ituC* (iturin) (Hofemeister et al., 2004). Compared with previous studies, the two highly protective strains F85 and T113 in this work contain more than 10 antibiotic biosynthesis genes, belonging to multiple types of antibiotics including non-ribosomal peptide synthetase and ribosomal peptide synthetase. Hence, F85 and T113 could produce a variety of antibiotics, and both have great potential in biocontrol research in the future.

## Conclusion

In summary, two new potential biocontrol strains against clubroot, *B. velezensis* F85 and *B. amyloliquefaciens* T113, were identified from *Brassica napus* rhizosphere. To our knowledge, this is the first report of a *B. velezensis* strain as a promising biocontrol agent against this disease. Future studies to elucidate biocontrol mechanism and field control efficacy of F85 and T113 are needed, and such information may lead to development of novel strategies to manage clubroot caused by *P. brassicae*.

## Data Avalability Statement

The original genome sequence data of strains F85 and T113 are available in GenBank (Accession SRR9925238 and SRR9925239).

## Author Contributions

LZ designed the experiments. MZ, YH, YL, TR, and HL performed the experimental work. MZ, LZ, JH, DJ, and TH analyzed the data. MZ, LZ, and TH wrote the manuscript.

## Conflict of Interest

The authors declare that the research was conducted in the absence of any commercial or financial relationships that could be construed as a potential conflict of interest.

## Footnotes

1 http://www.ncbi.nlm.nih.gov

2 www.i-sanger.com

3 http://ccb.jhu.edu/software/glimmer/index.shtml

4 http://lowelab.ucsc.edu/tRNAscan-SE/

5 http://www.vicbioinformatics.com/software.barrnap.shtml

## Supplementary Material

FIGURE S1 Distribution of base composition on clean reads of F85 and T113 genomic data. **(A)** Distribution of base composition on clean reads in F85. **(B)** Distribution of base composition on clean reads in T113. X axis represents base position along reads. Y axis represents base content percentage. Different bases are represented by different colors. As to high quality sequencing reads, A (adenine base) curve should be strictly overlapped with T (thymine base) curve, and G (guanine bsase) curve should be overlapped with C (cytosine base) curve according to the principle of complementary base pairing.

FIGURE S2 Distribution of base quality on clean reads of F85 and T113 genomic data. **(A)** Distribution of base quality on clean reads in F85. **(B)** Distribution of base quality on clean reads in T113. X axis represents base positions along reads. Y axis represents base quality value. The first half is Q-value distribution of reads at the first end of the double-end sequencing sequence, and Q-value distribution of the sequencing reads at the other end.

FIGURE S3 Inhibitory effect of F85 and T113 on the germination and viability of *P. brassicae* resting spores. **(A)** Germination of resting spore was inhibited by F85 or T113. After staining with orcein dye, resting spores without color were considered to be germinating whereas colored spores were considered to be non-germinated ones. G, germinated resting spores; N, non-germinated resting spores. Bar = 50 μm. **(B)** Viability of resting spores were reduced by F85 or T113. Colorless resting spores were active and dark blue resting spores were inactive. V, viable resting spores; I, inactive resting spores. Bar = 50 μm. Each experiment was repeated three times.

TABLE S1 Carbon source utilization pattern of F85 and T113 using BIOLOG GEN III system.

TABLE S2 Number of genes of F85 in category of GO annotation.

TABLE S3 Number of genes of T113 in category of GO annotation.

TABLE S4 Number of genes in category of KEGG pathway.
