# Supplementary figures and images for: Two New Biocontrol Agents Against Clubroot Caused by Plasmodiophora brassicae
Source: Front Microbiol. 2020 Jan 21;10:3099. doi: 10.3389/fmicb.2019.03099 (PMC6986203; doi:10.3389/fmicb.2019.03099)

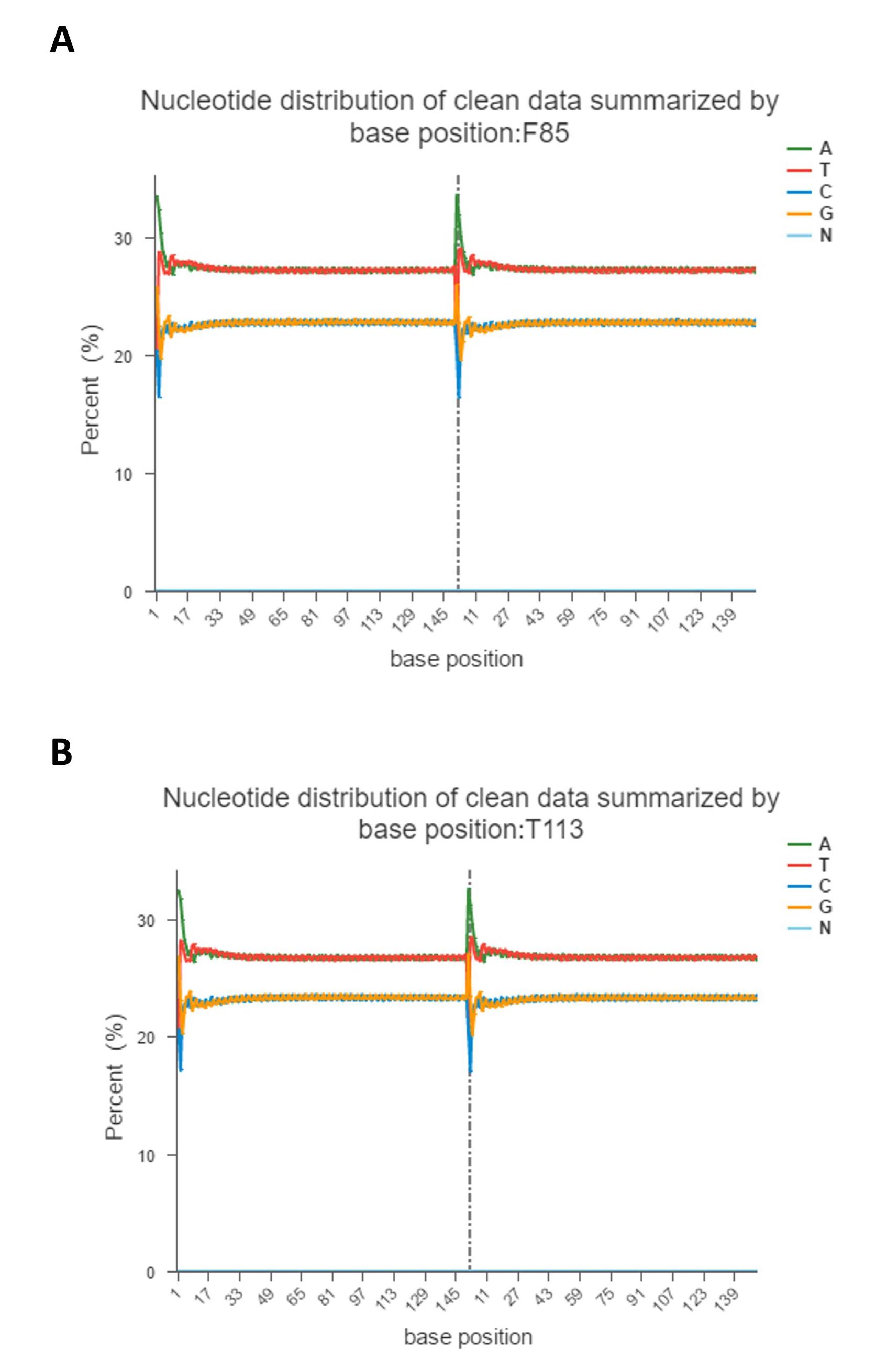

Supplement: FIGURE S1 — Distribution of base composition on clean reads of F85 and T113 genomic data. (A) Distribution of base composition on clean reads in F85. (B) Distribution of base composition on clean reads in T113. X axis represents base position along reads. Y axis represents base content percentage. Different bases are represented by different colors. As to high quality sequencing reads, A (adenine base) curve should be strictly overlapped with T (thymine base) curve, and G (guanine bsase) curve should be overlapped with C (cytosine base) curve according to the principle of complementary base pairing. [file Image_1.JPEG]

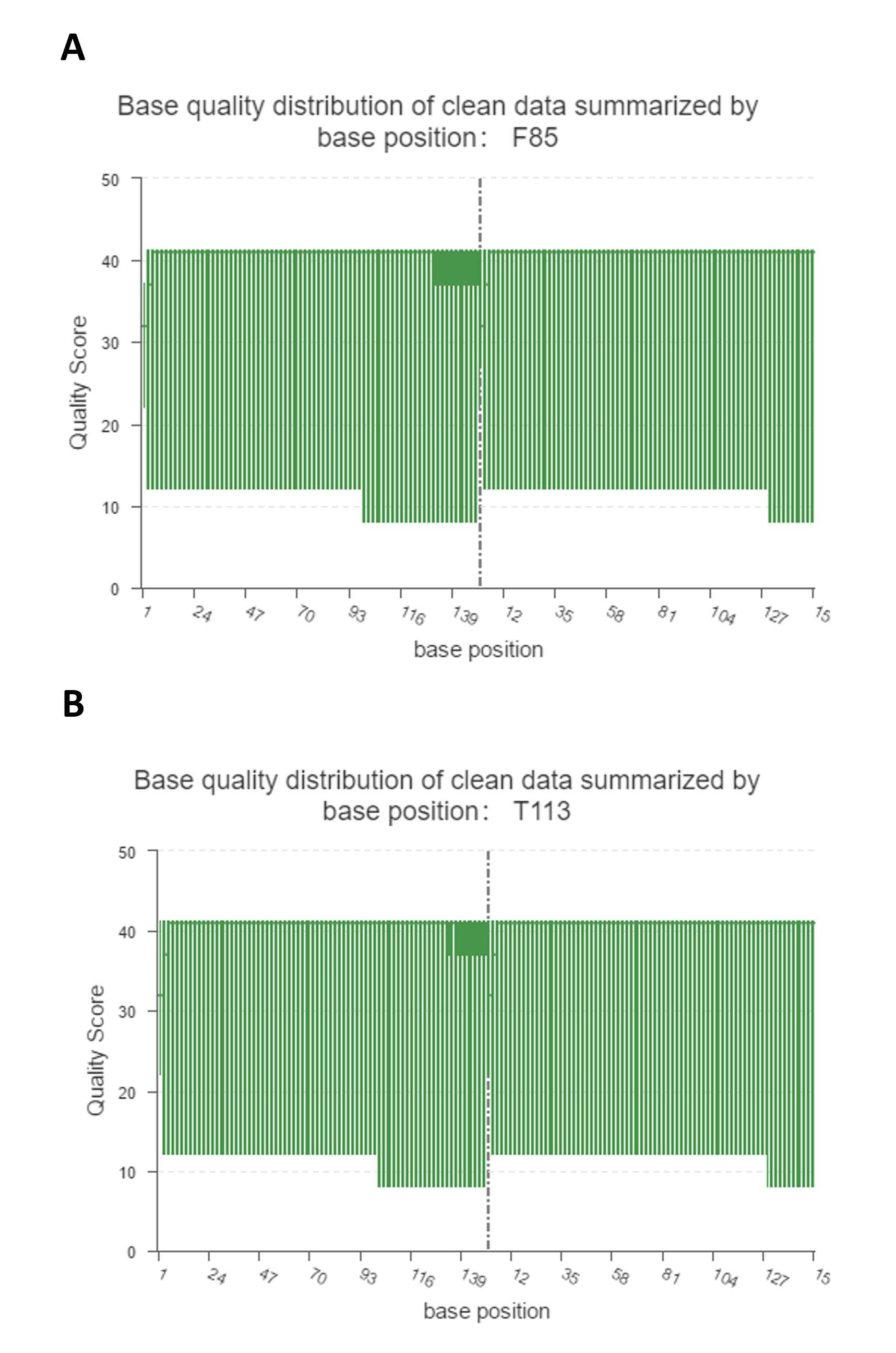

Supplement: FIGURE S2 — Distribution of base quality on clean reads of F85 and T113 genomic data. (A) Distribution of base quality on clean reads in F85. (B) Distribution of base quality on clean reads in T113. X axis represents base positions along reads. Y axis represents base quality value. The first half is Q-value distribution of reads at the first end of the double-end sequencing sequence, and Q-value distribution of the sequencing reads at the other end. [file Image_2.JPEG]

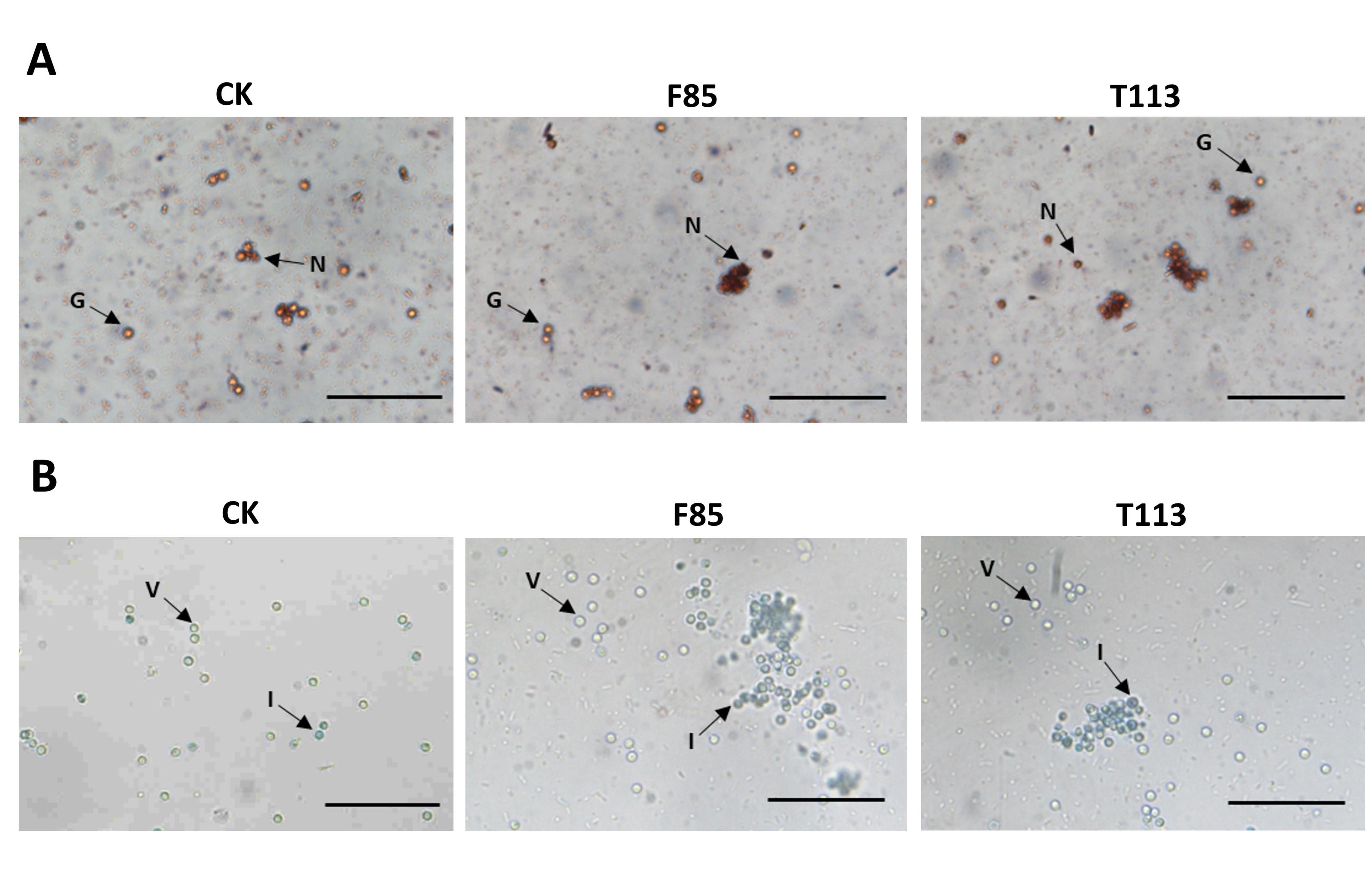

Supplement: FIGURE S3 — Inhibitory effect of F85 and T113 on the germination and viability of P. brassicae resting spores. (A) Germination of resting spore was inhibited by F85 or T113. After staining with orcein dye, resting spores without color were considered to be germinating whereas colored spores were considered to be non-germinated ones. G, germinated resting spores; N, non-germinated resting spores. Bar = 50 μm. (B) Viability of resting spores were reduced by F85 or T113. Colorless resting spores were active and dark blue resting spores were inactive. V, viable resting spores; I, inactive resting spores. Bar = 50 μm. Each experiment was repeated three times. [file Image_3.JPEG]
