# Supplementary material for: Two New Biocontrol Agents Against Clubroot Caused by Plasmodiophora brassicae
Source: Front Microbiol. 2020 Jan 21;10:3099. doi: 10.3389/fmicb.2019.03099 (PMC6986203; doi:10.3389/fmicb.2019.03099)
Supplement: TABLE S1 — Carbon source utilization pattern of F85 and T113 using BIOLOG GEN III system. [file Data_Sheet_1.docx]

**Table S1** Carbon source utilization pattern of F85 and T113 using BIOLOG GEN III system

| **No.** | **Nutrient** | **Reaction** | | **No.** | **Nutrient** | **Reaction** | | **No.** | **Nutrient** | **Reaction** | |
| --- | --- | --- | --- | --- | --- | --- | --- | --- | --- | --- | --- |
|  |  | **F85** | **T113** |  |  | **F85** | **T113** |  |  | **F85** | **T113** |
| A1 | water | - | - | C9 | Inosine | - | - | F5 | D-Glucuronic Acid | +/- | +/- |
| A2 | Dextrin | +/- | +/- | C10 | 1% Sodium Lactate | + | + | F6 | Glucuronamid e | +/- | +/- |
| A3 | D-Maltose | +/- | +/- | C11 | Fusidic Acid | - | - | F7 | Mucic Acid | - | - |
| A4 | D-Trehalose | +/- | +/- | C12 | D-Serine | - | - | F8 | Quinic Acid | +/- | + |
| A5 | D-Cellobiose | + | +/- | D1 | D-Sorbitol | + | + | F9 | D-Saccharic Acid | - | - |
| A6 | Gentiobiose | +/- | +/- | D2 | D-Mannitol | + | + | F10 | Vancomycin | - | - |
| A7 | Sucrose | + | + | D3 | D-Arabitol | - | - | F11 | Tetrazolium Violet | +/- | +/- |
| A8 | D-Turanose | +/- | - | D4 | myo-Inositol | + | + | F12 | Tetrazolium Blue | +/- | _ |
| A9 | Stachyose | +/- | - | D5 | Glycerol | + | + | G1 | p- Phenylacetic Acid | - | - |
| A10 | Positive Control | + | + | D6 | D-Glucose- 6-PO4 | +/- | +/- | G2 | Methyl Pyruvate | +/- | +/- |
| A11 | pH 6 | + | + | D7 | D-Fructose-6-PO4 | +/- | +/- | G3 | D- Acid Methyl Ester | - | - |
| A12 | pH 5 | + | + | D8 | D-Aspartic Acid | - | +/- | G4 | L-Lactic Acid | +/- | + |
| B1 | D-Raffinose | +/- | - | D9 | D-Serine | - | - | G5 | Citric Acid | + | + |
| B2 | α-D-Lactose | +/- | +/- | D10 | Troleandomycin | - | - | G6 | α- Glutaric Acid | - | - |
| B3 | D-Melibiose | +/- | +/- | D11 | Rifamycin SV | - | - | G7 | D-Malic Acid | - | - |
| B4 | β- D-Glucoside | + | +/- | D12 | Minocycline | - | - | G8 | L-Malic Acid | + | + |
| B5 | D-Salicin | +/- | +/- | E1 | Gelatin | +/- | +/- | G9 | Succinic Acid | - | - |
| B6 | N-D-Glucosamine | + | +/- | E2 | Glycyl-L-Prolin | +/- | +/- | G10 | Nalidixic Acid | +/- | - |
| B7 | N- D-Mannosamine | +/- | +/- | E3 | L-Alanine | + | + | G11 | Lithium Chloride | + | + |
| B8 | N-D-Galactosamine | - | - | E4 | L-Arginine | + | +/- | G12 | Potassium Tellurite | + | + |
| B9 | N-Neuraminic Acid | - | - | E5 | L-Aspartic Acid | + | + | H1 | Tween 40 | - | - |
| B10 | 1% NaCl | + | + | E6 | L-Glutamic Acid | + | + | H2 | γ-Amino-Butryric Acid | - | - |
| B11 | 4% NaCl | + | + | E7 | L-Histidine | _ | + | H3 | α-Hydroxy-Butyric Acid | +/- | - |
| B12 | 8% NaCl | + | + | E8 | L-Pyroglutamic Acid | - | - | H4 | β- -D-L Butyric Acid | + | - |
| C1 | α-D-Glucose | + | + | E9 | L-Serine | - | - | H5 | α-Keto-Butyric Acid | - | - |
| C2 | D-Mannose | + | + | E10 | Lincomycin | - | - | H6 | Acetoacetic Acid | +/- | - |
| C3 | D-Fructose | + | + | E11 | Guanidine HCl | + | + | H7 | Propionic Acid | - | - |
| C4 | D-Galactose | - | - | E12 | Niaproof 4 | - | - | H8 | Acetic Acid | - | - |
| C5 | 3-Methyl Glucose | +/- | - | F1 | Pectin | +/- | +/- | H9 | Formic Acid | - | +/- |
| C6 | D-Fucose | +/- | +/- | F2 | D-Galacturonic Acid | +/- | + | H10 | Aztreonam | - | +/- |
| C7 | L-Fucose | +/- | - | F3 | L-Galactonic Acid | + | + | H11 | Sodium Butyrate | +/- | + |
| C8 | L-Rhamnose | - | - | F4 | D-Gluconic Acid | +/- | +/- | H12 | Sodium Bromate | - | +/- |

**Table S2** Number of genes of F85 in category of GO annotation

| **Category** | **Function Description** | **GO ID (Level2)** | **Gene No.** |
| --- | --- | --- | --- |
| Biological process | metabolic process | GO:0008152 | 1338 |
|  | cellular process | GO:0009987 | 1119 |
|  | single-organism process | GO:0044699 | 899 |
|  | localization | GO:0051179 | 329 |
|  | biological regulation | GO:0065007 | 305 |
|  | regulation of biological process | GO:0050789 | 298 |
|  | response to stimulus | GO:0050896 | 163 |
|  | cellular component organization or biogenesis | GO:0071840 | 121 |
|  | developmental process | GO:0032502 | 84 |
|  | signaling | GO:0023052 | 61 |
|  | locomotion | GO:0040011 | 25 |
|  | detoxification | GO:0098754 | 22 |
|  | negative regulation of biological process | GO:0048519 | 18 |
|  | reproduction | GO:0000003 | 15 |
|  | multi-organism process | GO:0051704 | 10 |
|  | positive regulation of biological process | GO:0048518 | 6 |
|  | reproductive process | GO:0022414 | 5 |
|  | biological adhesion | GO:0022610 | 2 |
| Cellular component | membrane | GO:0016020 | 780 |
|  | membrane part | GO:0044425 | 738 |
|  | cell | GO:0005623 | 642 |
|  | cell part | GO:0044464 | 626 |
|  | macromolecular complex | GO:0032991 | 105 |
|  | organelle | GO:0043226 | 74 |
|  | organelle part | GO:0044422 | 39 |
|  | virion | GO:0019012 | 29 |
|  | virion part | GO:0044423 | 29 |
|  | extracellular region | GO:0005576 | 8 |
|  | nucleoid | GO:0009295 | 4 |
|  | supramolecular complex | GO:0099080 | 1 |
|  | other organism | GO:0044215 | 1 |
|  | other organism part | GO:0044217 | 1 |
|  | nutrient reservoir activity | GO:0045735 | 2 |
|  | catalytic activity | GO:0003824 | 1382 |
| Molecular function | binding | GO:0005488 | 948 |
|  | transporter activity | GO:0005215 | 206 |
|  | nucleic acid binding transcription factor activity | GO:0001071 | 116 |
|  | structural molecule activity | GO:0005198 | 42 |
|  | signal transducer activity | GO:0004871 | 34 |
|  | molecular transducer activity | GO:0060089 | 28 |
|  | transcription factor activity, protein binding | GO:0000988 | 23 |
|  | electron carrier activity | GO:0009055 | 22 |
|  | antioxidant activity | GO:0016209 | 22 |
|  | molecular function regulator | GO:0098772 | 3 |
|  | D-alanyl carrier activity | GO:0036370 | 1 |

**Table S3** Number of genes of T113 in category of GO annotation

| **Category** | **Function Description** | **GO ID (Level2)** | **Gene No.** |
| --- | --- | --- | --- |
| Biological process | metabolic process | GO:0008152 | 1361 |
|  | cellular process | GO:0009987 | 1132 |
|  | single-organism process | GO:0044699 | 940 |
|  | localization | GO:0051179 | 330 |
|  | biological regulation | GO:0065007 | 320 |
|  | regulation of biological process | GO:0050789 | 313 |
|  | response to stimulus | GO:0050896 | 176 |
|  | cellular component organization or biogenesis | GO:0071840 | 119 |
|  | developmental process | GO:0032502 | 86 |
|  | signaling | GO:0023052 | 69 |
|  | detoxification | GO:0098754 | 25 |
|  | locomotion | GO:0040011 | 23 |
|  | negative regulation of biological process | GO:0048519 | 18 |
|  | reproduction | GO:0000003 | 15 |
|  | multi-organism process | GO:0051704 | 11 |
|  | positive regulation of biological process | GO:0048518 | 5 |
|  | reproductive process | GO:0022414 | 5 |
| Cellular component | membrane | GO:0016020 | 794 |
|  | membrane part | GO:0044425 | 757 |
|  | cell | GO:0005623 | 655 |
|  | cell part | GO:0044464 | 641 |
|  | macromolecular complex | GO:0032991 | 102 |
|  | organelle | GO:0043226 | 71 |
|  | organelle part | GO:0044422 | 38 |
|  | virion part | GO:0044423 | 24 |
|  | virion | GO:0019012 | 24 |
|  | nucleoid | GO:0009295 | 5 |
|  | extracellular region | GO:0005576 | 4 |
| Molecular function | catalytic activity | GO:0003824 | 1409 |
|  | binding | GO:0005488 | 973 |
|  | transporter activity | GO:0005215 | 210 |
|  | nucleic acid binding transcription factor activity | GO:0001071 | 117 |
|  | structural molecule activity | GO:0005198 | 42 |
|  | signal transducer activity | GO:0004871 | 37 |
|  | molecular transducer activity | GO:0060089 | 31 |
|  | antioxidant activity | GO:0016209 | 25 |
|  | electron carrier activity | GO:0009055 | 24 |
|  | transcription factor activity, protein binding | GO:0000988 | 23 |
|  | molecular function regulator | GO:0098772 | 3 |
|  | nutrient reservoir activity | GO:0045735 | 1 |
|  | D-alanyl carrier activity | GO:0036370 | 1 |

**Table S4** Number of genes in category of KEGG pathway

| **First Category** | **Second Category** | **Gene No. of F85** | **Gene No. of T113** |
| --- | --- | --- | --- |
| Cellular Processes | Cell motility | 48 | 48 |
|  | Cell growth and death | 20 | 19 |
|  | Cellular community - prokaryotes | 79 | 83 |
|  | Transport and catabolism | 13 | 13 |
| Metabolism | Biosynthesis of other secondary metabolites | 30 | 31 |
|  | Lipid metabolism | 82 | 78 |
|  | Global and overview maps | 212 | 210 |
|  | Xenobiotics biodegradation and metabolism | 33 | 32 |
|  | Metabolism of terpenoids and polyketides | 45 | 50 |
|  | Carbohydrate metabolism | 235 | 240 |
|  | Metabolism of cofactors and vitamins | 159 | 157 |
|  | Metabolism of other amino acids | 47 | 46 |
|  | Amino acid metabolism | 203 | 205 |
|  | Energy metabolism | 113 | 118 |
|  | Nucleotide metabolism | 96 | 88 |
|  | Glycan biosynthesis and metabolism | 32 | 32 |
| Human Diseases | Cancers: Overview | 12 | 13 |
|  | Infectious diseases: Bacterial | 17 | 17 |
|  | Endocrine and metabolic diseases | 3 | 8 |
|  | Cardiovascular diseases | 4 | 3 |
|  | Neurodegenerative diseases | 8 | 4 |
|  | Cancers: Specific types | 2 | 2 |
|  | Infectious diseases: Viral | 1 | 1 |
|  | Drug resistance: Antimicrobial | 30 | 30 |
|  | Drug resistance: Antineoplastic | 6 | 7 |
|  | Immune diseases | 1 | 1 |
|  | Infectious diseases: Parasitic | 1 | 1 |
| Genetic Information Processing | Translation | 66 | 67 |
|  | Replication and repair | 55 | 49 |
|  | Folding, sorting and degradation | 48 | 48 |
|  | Transcription | 5 | 5 |
| Organismal Systems | Immune system | 3 | 3 |
|  | Endocrine system | 13 | 13 |
|  | Aging | 9 | 9 |
|  | Excretory system | 2 | 2 |
|  | Digestive system | 2 | 2 |
|  | Nervous system | 3 | 3 |
|  | Environmental adaptation | 3 | 3 |
| Environmental Information Processing | Signal transduction | 128 | 131 |
|  | Membrane transport | 154 | 154 |
|  | Signaling molecules and interaction | 1 | 1 |
